# Beyond Likeability: Investigating Social Interactions with Artificial Agents and Objective Metrics

**DOI:** 10.3389/fpsyg.2017.01662

**Published:** 2017-09-22

**Authors:** Sebastian Loth

**Affiliations:** Social Cognitive Systems and Psycholinguistics, Centre of Excellence on Cognitive Interaction Technology (CITEC), Faculty of Technology, Bielefeld University Bielefeld, Germany

**Keywords:** virtual agents, social signals, interaction design, gaze aversion, social eye gaze, dialogue capability, facial animation

Numerous studies have investigated whether additional abilities of artificial agents improve the interaction with their users. Typically, a specific capability was added to a baseline system and participants interacted with both versions. Questionnaires (e.g., GOODSPEED, Bartneck et al., 2009) or time-related metrics combined with a questionnaire (e.g., PARADISE, Walker et al., 2000) were then evaluated. In most published work, the new capability demonstrably improved the user ratings. For example, agents with the capability to praise (Fasola and Mataric, 2012), err (Salem et al., 2013), and blink pleasantly (Takashima et al., 2008) were more likeable than their counterparts without this capability. However, subjective ratings can be unreliable (Cahill, 2009; Belz and Kow, 2010) and hardly address questions beyond likeability, e.g., whether an action is perceived as social or task-oriented, and when and why an agent is more or less comprehensible. But investigating human social behavior with controlled and ecologically valid experiments is notoriously difficult given the variance in natural interactions (e.g., Bergmann et al., 2010; Sciutti et al., 2015). Artificial agents can precisely reproduce interactive behavior in real-time but are still rarely used for investigations beyond likeability.

Conrad et al. (2015) used subjective ratings and objective metrics (gaze behavior, response accuracy, number of clarification questions and backchannels) for investigating how the users' comprehension of straightforward and complicated survey questions^1^ was affected by the virtual agent's facial expressiveness^2^ and dialogue capability^3^. As expected, the subjective ratings improved with more advanced agent capabilities. Objective metrics such as response accuracy and eye tracking data provided further insights, e.g., whether interviewees avoid eye contact with their interviewer (gaze aversion). Research in human-human interaction indicated that gaze aversion was not social behavior but that it reduced distraction and directed all the interviewee's cognitive resources toward answering the question (Glenberg et al., 1998; Doherty-Sneddon et al., 2002; Doherty-Sneddon and Phelps, 2005). But the interviewees' gaze patterns in Conrad et al's (2015) study were independent of the question's difficulty if the agent had high dialogue capabilities. This implies a social component in gaze aversion that cannot be ruled out by the original studies that used (a) human questioners such that gaze aversion could be social (Glenberg et al., 1998; Doherty-Sneddon et al., 2002), (b) no questioner such that truly social gaze behavior was impossible to observe (Glenberg et al., 1998), or (c) memory tasks (Glenberg et al., 1998; Doherty-Sneddon and Phelps, 2005) that are sensitive to any intervening information such as faces (Posner and Konick, 1966; Dale, 1973; West, 1999; de Fockert et al., 2001). A social component in gaze aversion is not surprising given the social nature of other behaviors. For example, facial expressions of surprise were more pronounced if other humans were present in the room (Schützwohl and Reisenzein, 2012). Even the electrodermal activity of truth tellers and deceivers differed only if they believed their virtual interviewer was controlled by human but not by artificial intelligence (Ströfer et al., 2016). Thus, gaze aversion appears to be at least partly social. Conrad et al's (2015) findings illustrate that objective metrics in HMI reveal new and additional insights (cf. Zarrieß et al., 2015) providing a valuable tool for understanding social interaction.

Designing artificial agents driven by likeability tends to accumulate capabilities regardless of their contribution to the interaction. First, (likeable) capabilities contribute differently to an interaction and do not necessarily increase an artificial agent's comprehensibility. Conrad et al. (2015) tested the effect of each capability on their participants' behavior. For example, high dialogue capabilities produced the same response accuracy irrespectively of the agent's facial expressiveness. On the other hand, facial expressiveness influenced the participants' back-channeling behavior which could smoothen the interaction (cf. Clark and Brennan, 1991; Clark and Krych, 2004). Secondly, simply combining likeable behaviors does not necessarily result in a human-like, comprehensible artificial agent. For example, humans have difficulty integrating human beat gestures and speech if enacted by a robot (Bremner and Leonards, 2015). Also, averaging across behaviors of individuals may result in an unintelligible chimera whereas humans prefer the behavior of one individual (Bergmann et al., 2010; Kelly et al., 2010). Arguably, a fictional character (or artificial agent) gains appeal by focussing on essential communicative behaviors that form a coherent, expressive and comprehensible individual (Thomas and Johnston, 1995). Thirdly, not being perceived as human may not be likeable but advantageous because users would assume an automatic agent operates consistently rather than having bad intentions or preferring someone over someone else. Also, this intuitively differentiates between humans and artificial agents (cf. Stein and Ohler, 2017). In contrast to being all human-like, using human cognitive principles for maximizing an agent's capability to interact with its users is highly desirable. Thus, objective metrics and insightfully designed experiments are more important than likeable capabilities.

In sum, Conrad et al. (2015) estimated the communicative relevance of an artificial agent's social behaviors given a specific task with objective metrics such as response accuracy and gaze patterns. Their study demonstrated that the difficult research in social interaction can benefit from artificial agents if combined with insightful, reliable, replicable and objective dependent metrics.

## Author contributions

The author confirms being the sole contributor of this work and approved it for publication.

### Conflict of interest statement

The author declares that the research was conducted in the absence of any commercial or financial relationships that could be construed as a potential conflict of interest.

## Footnotes

^1^Questions from the US current population survey (monthly survey of 60,000 households estimating, e.g., unemployment rate, see United States Census Bureau, 2016) were “complicated” if they were ambiguous (e.g., is a floor lamp a piece of furniture or an electrical appliance) and likely triggered clarification questions.

^2^Facial animation was either low (head and face did not move, the eyes did not blink and the mouth was only opened and closed indicating whether the agent was speaking) or high (21 channel-motion tracking of a human face, appropriate lip movements, blinking behavior at all times during the interview).

^3^Dialogue capability was either low (the agent responded to user questions with a neutral probe or repeated the interview question) or high (the agent tried to provide helpful answers such as clarifying terms). In both conditions, the agent read out the interview questions and repeated them if needed.
